# GABAergic and glutamatergic mechanisms of cortical excitability shape motor resonance during action observation as measured by transcranial magnetic stimulation

**DOI:** 10.3389/fnhum.2026.1746409

**Published:** 2026-03-23

**Authors:** Aynur Ragimova, Madina Imanaeva, Carlos Nieto-Doval, Alicia N. Vorobiova, Victoria Moiseeva, Matteo Feurra

**Affiliations:** 1 Centre for Cognition and Decision Making, Institute for Cognitive Neuroscience, HSE University, Moscow, Russia; 2 Research Center of Neurology, Moscow, Russia; 3 Department of Psychology, HSE University, Moscow, Russia

**Keywords:** action observation, intracortical facilitation, mirror neuron system, motor cortex excitability, motor resonance, short-interval intracortical inhibition, transcranial magnetic stimulation

## Abstract

**Background:**

Motor resonance reflects the activation of the motor system during the observation of others’ actions and is considered a physiological marker of the human mirror neuron system (MNS). This study investigated whether the excitatory and inhibitory balance in the primary motor cortex, driven by glutamatergic and GABAergic mechanisms, is linked to the strength of muscle-specific responses during action observation (AO).

**Objectives:**

To determine whether cortical excitation-inhibition balance relates to muscle-specific motor resonance during AO.

**Methods:**

Using single pulse Transcranial Magnetic Stimulation (spTMS), we measured motor evoked potentials (MEPs) from the first dorsal interosseous (FDI) and abductor digiti minimi (ADM) during an AO task involving videos of index and little finger movements. In addition, corticospinal excitability indices were assessed using single-pulse (SP), intracortical facilitation (ICF), and short-interval intracortical inhibition (SICI) protocols before and after AO. ICF reflects glutamatergic facilitation, while SICI indexes GABA-A mediated inhibition.

**Results:**

Across excitability protocols ICF consistently elicited the largest MEPs, followed by SP and SICI. After AO, MEPs increased in both ICF and SP protocols, whereas SICI showed reduced inhibitory effects. During AO, SP-induced MEPs in the FDI showed reduced corticospinal excitability during little finger movement observation, whereas ADM displayed increased excitability during neutral and little finger movement conditions. Correlation analyses indicated that both pre- and post-AO excitability measures correlated with task-related MEP modulation. Notably, higher post-AO ICF in ADM was associated with stronger ADM responses during observation of little finger movements. Greater pre-AO SICI in ADM was associated with stronger suppression of SP-induced ADM MEPs during observation of index finger movements. Additionally, FDI ICF, both before and after AO, correlated with SP-induced FDI MEP responses during index finger observation.

**Conclusion:**

Taken together, these results indicate that motor resonance arises from the interplay between excitatory and inhibitory processes in the motor cortex and that individual differences in motor resonance are constrained by stable excitatory–inhibitory properties indexed by ICF and SICI. These insights into cortical excitation-inhibition dynamics provide a neurophysiological basis for individualized interventions in neuropsychiatric and motor disorders characterized by disrupted motor resonance or corticospinal excitability.

## Introduction

Motor resonance refers to changes in motor system excitability during action observation (AO) and is considered fundamental to social interaction, imitation, and possibly language acquisition (Gallese, 2008; Catmur et al., 2011; Iacoboni, 2009; Nieto-Doval et al., 2025; Rizzolatti and Craighero, 2004). It reflects the automatic mapping of observed actions onto the observer’s motor system, even in the absence of overt movement. Although this phenomenon is often discussed in the context of the mirror neuron system (MNS), it is important to note that in human research, MNS activity is typically inferred from indirect measures, such as changes in motor evoked potentials (MEPs) induced by single pulse transcranial magnetic stimulation (spTMS) over primary the motor cortex (M1) during AO tasks (Fadiga et al., 2005; Uithol et al., 2011; Bolzoni et al., 2023; Barchiesi and Cattaneo, 2015). Transcranial magnetic stimulation (TMS) has been widely used to probe motor resonance by measuring MEPs elicited during AO (Barchiesi and Cattaneo, 2012; Errante and Fogassi, 2020; Nieto-Doval et al., 2023). While these MEP changes reflect sensorimotor reactivity to observed actions, the underlying neurochemical mechanisms remain unclear. In particular, the balance between cortical excitation and inhibition, primarily mediated by glutamatergic and GABAergic circuits, may play a critical role in modulating the strength and direction of motor resonance responses. Two paired-pulse TMS protocols, intracortical facilitation (ICF) and short-interval intracortical inhibition (SICI), are well-established measures of corticospinal excitability, indexing glutamatergic NMDA receptor activity and GABA-A mediated inhibition, respectively (Kujirai et al., 1993; Rothwell et al., 2009; Chen et al., 2018; Cash et al., 2017). These protocols reliably reveal changes in intracortical excitability associated with motor learning, fatigue, and neurological state (Hunter et al., 2016), highlighting their relevance for studying plasticity and adaptive motor responses.

Importantly, corticospinal excitability measures such as ICF and SICI show substantial interindividual variability, which has been linked to anatomical properties of the corticospinal tract, supporting their interpretation as biologically meaningful individual measures (Betti et al., 2022). The primary aim of this study was to determine whether individual measures of ICF and SICI correlate with motor resonance effects during an AO task involving index and little finger intransitive abduction movements and a non-moving hand (neutral condition). Specifically, we hypothesized that cortical excitability indices (ICF and SICI) recorded at rest prior to observing actions would correlate individual differences in motor resonance, as reflected in the amplitude of MEPs elicited during observation of finger movements. We anticipated that higher levels of ICF (indicative of stronger glutamatergic facilitation) and lower levels of SICI (suggestive of reduced GABAergic inhibition) would correlate with stronger motor resonance effects, providing evidence that stable neurochemical traits underlie individual variability in motor system responsiveness. As a critical control measure and secondary objective, we also assessed cortical excitability (ICF and SICI) after the AO task. This allowed us to verify whether the observed correlations remained stable or were influenced by the task itself and to characterize session-wide changes in corticospinal excitability across the experiment.

Clarifying the relationship between cortical excitability and motor resonance would significantly deepen our understanding of the neurochemical mechanisms underpinning action-perception coupling. Moreover, it could inform targeted therapeutic strategies for clinical populations characterized by excitatory-inhibitory imbalances and impaired sensorimotor integration.

## Methods

### Subjects

Twenty-nine right-handed volunteers (14 females, age range: 19–29 years, mean age: 22 years) participated in the study. Handedness was assessed by self-report based on habitual hand use. All participants reported no personal or family history of neurological or psychiatric disorders and abstained from alcohol, drugs, caffeine, and theine consumption for at least 4 h prior to each session. Written informed consent was obtained from all participants, who received financial compensation for their time. The study was conducted in accordance with the Declaration of Helsinki and was approved by the Ethical Committee of the National Research University Higher School of Economics, Moscow No. 80(2).

During the experiment, participants sat comfortably in a reclining chair with their head in a vertical position and their right arm relaxed. The elbow was positioned at approximately 90 degrees, ensuring that the hand remained parallel to the presentation screen. This posture was used to minimize potential confounding effects of spatial alignment between the observer’s and observed actions (Alaerts et al., 2009). Participants were instructed to avoid any hand movements throughout the experiment.

#### Transcranial magnetic stimulation

Neuronavigated transcranial magnetic stimulation (TMS) was applied to the left primary motor cortex (M1) using a MagPro X100 stimulator and coils C-B60 and C-B85 (MagVenture, Farum, Denmark). Precise coil placement was ensured using a frameless neuronavigation system (Localite TMS Navigator, Localite GmbH, Sankt Augustin, Germany) based on individual T1-weighted MRI scans, allowing accurate control of coil position and orientation and reducing interindividual anatomical variability across the experimental session. The optimal stimulation site (hotspot) was identified by exploring the left M1 and selecting a single coil position that reliably elicited stable MEPs in both the first dorsal interosseous (FDI) and abductor digiti minimi (ADM) muscles, with ADM amplitudes approximately two-thirds of those recorded in FDI (Nieto-Doval et al., 2025). A shared hotspot was intentionally used to ensure identical stimulation geometry, intensity, and coil orientation across muscles and across all stimulation protocols, thereby allowing direct within-subject comparisons of corticospinal excitability modulation during AO. We acknowledge that the use of a shared hotspot represents a methodological trade-off, as it is suboptimal for selectively targeting the cortical representations of individual hand muscles. This approach may contribute to differences in absolute MEP amplitudes between FDI and ADM and likely underlies the observed main effect of Muscle. Therefore, absolute differences between muscles should be interpreted with caution, and conclusions are primarily supported by within-muscle, within-condition comparisons.

Following manual identification of the hotspot using the C-B60 coil, stimulation continued with a C-B85 coil, which has the same butterfly shape and 75 mm diameter as the C-B60 coil, and was mounted on a robotic arm (Axilum TMS Cobot System; Brainbox Ltd., United Kingdom). The C-B60 coil was used during the neuronavigation phase to manually explore the motor cortex and identify the optimal motor hotspot, as its freehand handling allows fine spatial adjustments on a pulse-by-pulse basis. Once the hotspot was identified, stimulation was delivered using a C-B85 coil mounted on a robotic arm. Both coils generate identical magnetic fields. The Cobot system maintained consistent neuronavigation and preserved the hotspot position, ensuring precise stimulation throughout the experiment. The resting motor threshold (rMT) was determined using a staircase procedure until the minimum stimulation intensity evoking MEPs with at least 50 μV peak-to-peak amplitude in 50% of 10 consecutive pulses was identified (Rossini et al., 1994). The TMS intensity during experimental tasks was set at 120% of the rMT in the left (dominant) hemisphere. Surface electromyography (EMG) activity was recorded from the right FDI and ADM muscles using disposable adhesive surface electrodes (EB Neuro S.p. A., Florence, Italy) and a BrainAmp DC amplifier (Brain Products GmbH, Munich, Germany) with a sampling rate of 5 kHz.

#### Experimental procedure

**Figure 1:**
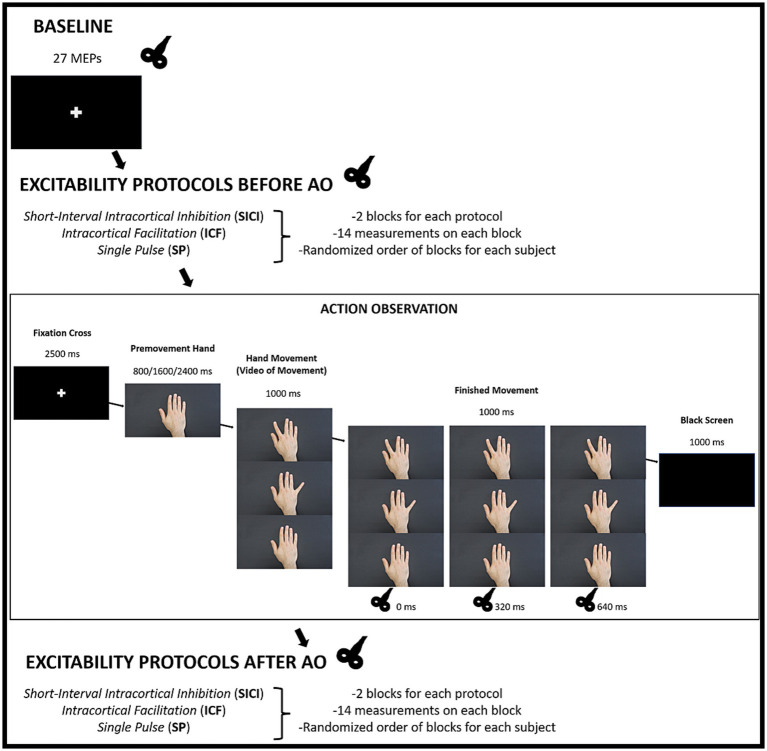
Stimulus presentation protocol using video recordings of full movements, with post-movement phase TMS stimulation.

Following identification of the stimulation hotspot and determination of the resting motor threshold (rMT), participants were seated comfortably with a presentation screen positioned in front of them. The experimental protocol began with a Baseline (resting state), during which participants viewed a fixation cross on a black screen while single-pulse TMS was applied. During this Baseline, 27 MEPs were collected and later used as the reference for normalization of all subsequent measures (Figure 1). After Baseline, cortical excitability was assessed through three stimulation protocols administered in a randomized order: intracortical facilitation (ICF), short-interval intracortical inhibition (SICI), and single-pulse (SP). Participants then performed the AO task, in which they observed index finger, little finger, or neutral hand movements while single-pulse TMS was delivered. Finally, the same three excitability protocols (ICF, SICI, SP) were repeated in randomized order to provide post-task control measures (Figure 1).

In total, 357 TMS pulses were delivered across all sections, targeting the cortical representation of both the first dorsal interosseous (FDI) and abductor digiti minimi (ADM) muscles. Visual stimuli were presented using E-Prime 3.0 software (Psychology Software Tools, Pittsburgh, PA), with TMS pulses delivered at randomized intervals and controlled via TTL markers through the Parallel Port.

Each experimental session lasted approximately 150 min in total, including participant setup, neuronavigation and hotspot localization, baseline recordings, paired-pulse excitability measures, and the AO task. Short breaks were provided throughout the session to minimize discomfort and fatigue. Participants remained seated in a relaxed position for the entire duration of the experiment, and no overt signs of fatigue were reported.

Brief pauses of approximately 1–2 min were introduced between each excitability protocol block. Longer breaks of approximately 3–4 min were provided between excitability protocol blocks (pre- and post-action observation) and the AO task, allowing sufficient time for protocol adjustments and participant rest.

#### Cortical excitability measures

Cortical excitability was assessed using short-interval intracortical inhibition (SICI), intracortical facilitation (ICF), and single-pulse (SP) stimulation protocols. Paired-pulse TMS followed standard parameters, with SICI at an interstimulus interval (ISI) of 2 ms and ICF at 12 ms, both within canonical windows (SICI ~1–5 ms; ICF ~ 10–15 ms) (Kujirai et al., 1993; Ni et al., 2007; Chen et al., 1998; Chen et al., 2018; Van den Bos et al., 2018). Specifically, SICI and ICF consisted of a subthreshold conditioning pulse followed by a suprathreshold test pulse delivered over the left M1 at 80 and 120% of the rMT, respectively. The inclusion of the SP condition provided an internal reference for corticospinal excitability within the same randomized block structure, allowing the effects of ICF and SICI to be interpreted relative to single-pulse responses. Single-pulse stimulation was delivered at 120% rMT. Each protocol (SICI, ICF, SP) was executed in two randomly administered blocks, with each block comprising 14 pulses, resulting in a total of 84 pulses, before and after the AO task. The inter-trial interval varied randomly between 3 and 7 s (Figure 1).

#### Action observation task

The action observation (AO) task consisted of 162 randomized trials, with 54 trials for each movement condition (index finger, little finger, and neutral hand). Each trial began with the presentation of a white fixation cross on a black background for 2,500 ms. This was followed by a static image of a hand in a neutral position, displayed for a randomized duration of 800, 1,600, or 2,400 ms in order to reduce stimulus predictability. The static hand image then transitioned into one of three video-recorded movements lasting 1,000 ms: abduction of the index finger, abduction of the little finger, or a continuation of the neutral hand display without movement. Each movement type was presented in a fully randomized order across the 54 trials assigned to that condition.

After the video, a static image of the final hand position was shown for 1,000 ms (post-movement phase). Single-pulse TMS was delivered at randomized intervals of 0, 320, or 640 ms (Catmur et al., 2007) from the onset of this post-movement phase. These stimulation timings were selected based on our previous work (Nieto-Doval et al., 2025), in which TMS was delivered during multiple phases of action observation (movement onset, mid-movement, static transition, and post-movement) to identify the epoch producing the most robust motor resonance. Because post-movement stimulation yielded the strongest and most reliable corticospinal modulation, the present study focused on this window. A total of 54 pulses were delivered at each timing condition. Each trial concluded with a black screen displayed for 1,000 ms (Figure 1).

#### Data analysis

A 10-Hz high-pass filter and a notch filter (50 Hz) to remove power-line noise were applied during the sessions for online visualization.

After filtering, trials with MEPs contaminated by artifacts, excessive latency, jitter, or visible muscular activity (as described above) were excluded from further analysis. For each valid trial, MEP amplitudes were extracted within a 20–35 ms post-stimulus window. The peak-to-peak amplitude of the MEP was calculated offline. MEPs with amplitudes below 50 μV were discarded.

Following artifact rejection, the raw MEP amplitudes from the first dorsal interosseous (FDI) and abductor digiti minimi (ADM) muscles were averaged for each condition, taking into account stimulus type and TMS timing. These averaged values were then normalized to the Baseline (resting state prior to the excitability protocols and the AO task), as percentage changes in the mean peak-to-peak amplitude of the Baseline (100%) for both FDI and ADM muscles (Rossini et al., 1999; Feurra et al., 2019). Normalization controlled for inter-subject variability in absolute MEP size.

Outlier detection was performed separately for the excitability protocols (SICI, ICF, SP) and the AO task. Two participants were excluded from further analysis because more than one-third of their normalized values were identified as outliers (i.e.: more than 2 standard deviations). For the remaining dataset, isolated outlier values were replaced with the mean amplitude of the corresponding muscle and condition. The mean replacement was applied only to isolated outlier values after participant-level exclusion and was used to maintain balanced within-subject conditions following artifact rejection.

### Statistical analysis

After preprocessing and adjustments, we conducted two separate three-way repeated-measures ANOVAs. The first ANOVA examined excitability protocols, with the within-subject factors Excitability (SICI, ICF, SP), Muscle (FDI, ADM), and Time (pre- vs. post-AO task). The second ANOVA focused on the action observation (AO) task, with the factors Movement (index finger, little finger, neutral), Muscle (FDI, ADM), and Stimulation Timing (0, 320, 640 ms). When significant interactions were identified, we performed Bonferroni-corrected pairwise comparisons. This correction was chosen to maintain the overall error rate at *α* = 0.05 while retaining sensitivity in line with the exploratory nature of the study. If Mauchly’s test of sphericity was violated (*p* < 0.05), we applied the Greenhouse–Geisser correction.

In addition, we conducted Pearson correlation analyses to test whether excitability indices correlated with motor responses during action observation. Specifically, we examined the relationships between MEPs recorded during AO (index and little finger movements, in ADM and FDI muscles) and excitability measures obtained with ICF and SICI protocols at rest, both before and after the AO task. This allowed us to determine whether glutamatergic facilitation (ICF) and GABAergic inhibition (SICI) were associated with muscle-specific excitability patterns during action observation.

## Results

### Excitability

A three-way repeated measures ANOVA was performed on mean motor evoked potentials (MEPs) normalized to Baseline, with factors Excitability protocol (SICI, ICF, SP), Muscle (FDI, ADM), and Time (pre- vs. post-AO). The analysis revealed a significant Excitability × Muscle interaction [*F*(1.557, 40.475) = 9.67, MSE = 20589.54, *p* = 0.001, η^2^ = 0.27]. In addition, there were significant main effects of Excitability [*F*(1.39, 46.134) = 109.99, MSE = 460673.45, *p* < 0.001, η^2^ = 0.81], Muscle [*F*(1, 26) = 14.43, MSE = 40283.03, *p* = 0.001, η^2^ = 0.36], and Time [*F*(1, 26) = 7.43, MSE = 18648.09, *p* = 0.011, η^2^ = 0.22].

**Figure 2:**
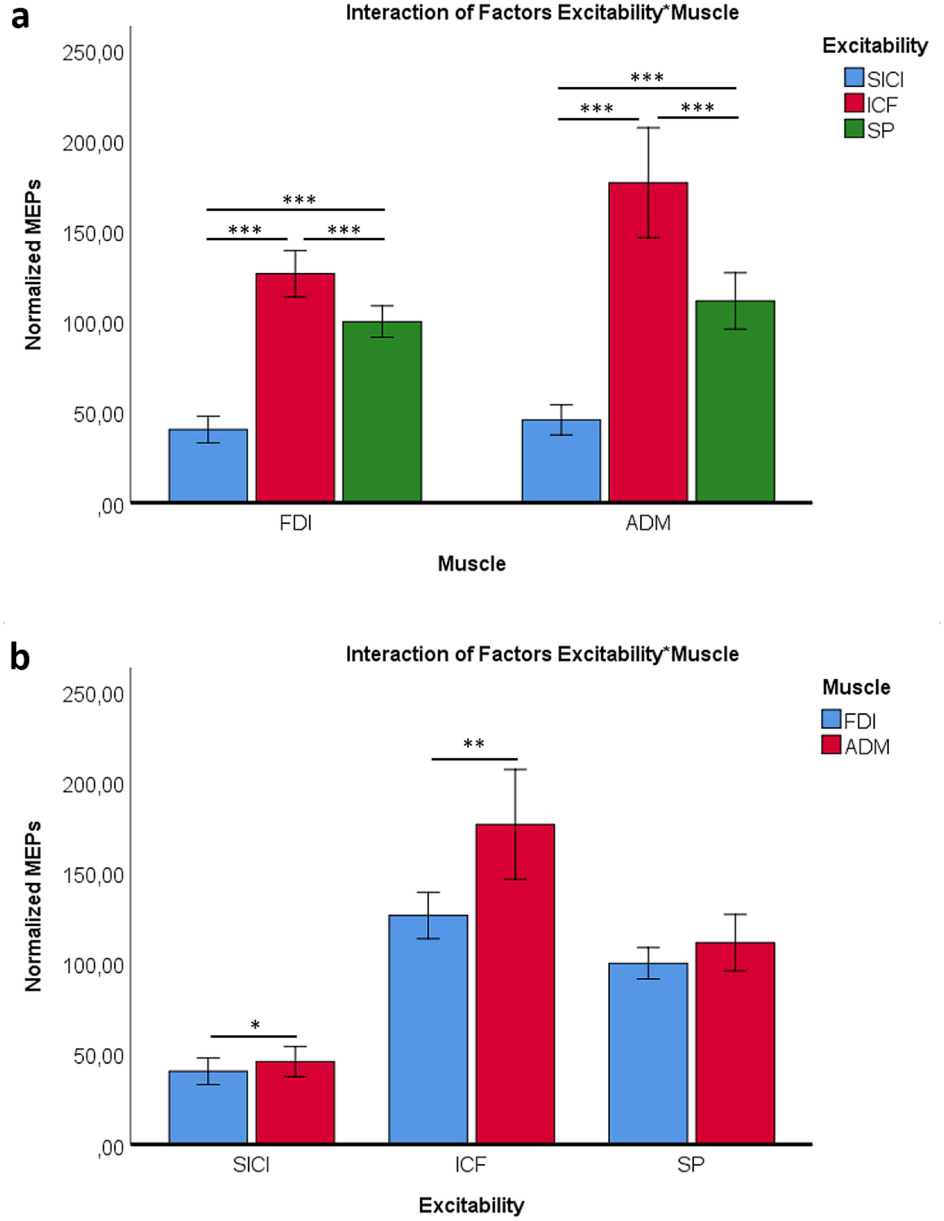
Interaction between excitability protocol (SICI, ICF, SP) and muscle (ADM, FDI), including pre- and post-AO values. **(a)** Mean MEP amplitudes for ADM and FDI across excitability protocols (SICI = inhibitory, ICF = facilitatory, SP = single-pulse control). **(b)** Comparison of ADM and FDI responses within each excitability protocol. *p < 0.05; **p < 0.01; ***p < 0.001.

The interaction between Excitability and Muscle showed consistent patterns, with both muscles exhibiting significant differences across protocols (ICF > SP, *p* < 0.001; ICF > SICI, *p* < 0.001; SP > SICI, *p* < 0.001). These effects were robust across protocols and muscles. The results confirm that SICI significantly suppressed MEPs in both ADM and FDI, whereas ICF facilitated them. The SP condition served as an internal control. Figure 2a illustrates these distinct inhibitory and facilitatory effects. Notably, the ADM muscle showed larger MEPs than the FDI for both ICF (*p* = 0.028) and SICI (*p* = 0.001) (Figure 2b).

**Figure 3:**
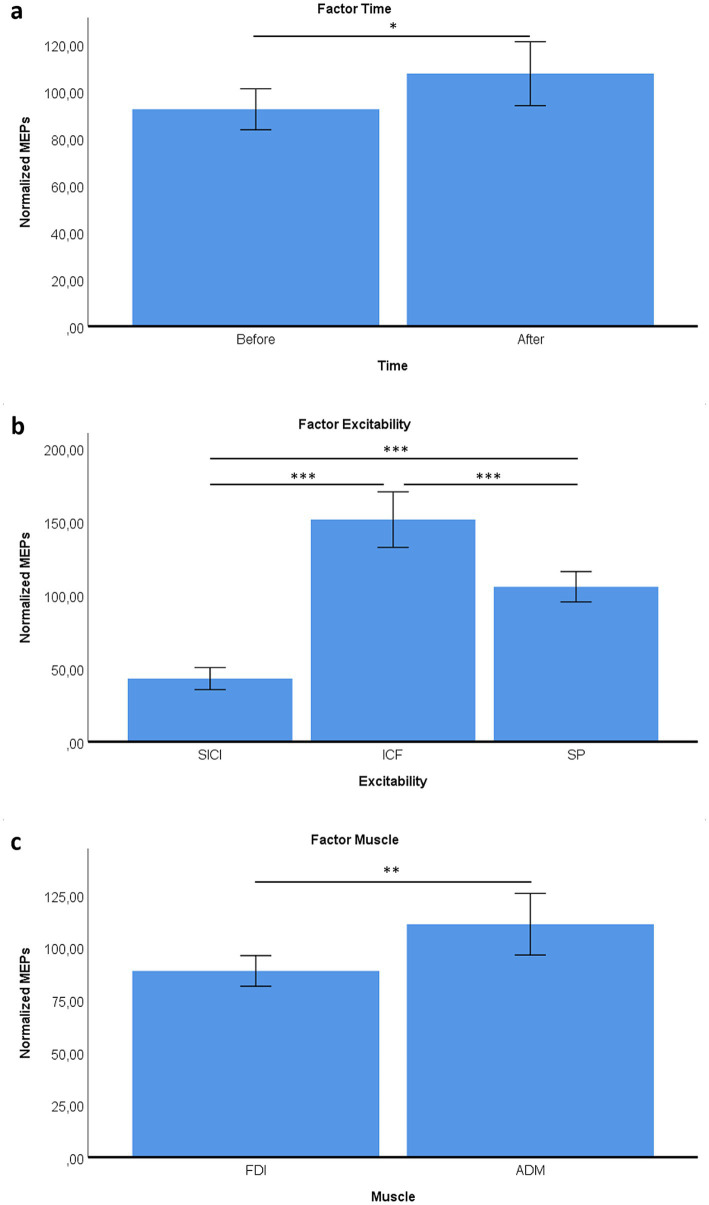
Effects of time, protocol, and muscle on cortical excitability. **(a)** Normalized MEP amplitudes were significantly higher after the AO task compared to before. **(b)** Comparison across excitability protocols (SICI = inhibitory, ICF = facilitatory, SP = single-pulse control) showing ICF > SP > SICI. **(c)** Muscle-specific differences, with ADM exhibiting greater MEP amplitudes than FDI. *p < 0.05; **p < 0.01; ***p < 0.001.

With respect to Time, normalized MEP values differed between pre- and post-AO measurements (*p* = 0.011), reflecting changes relative to the pre-task baseline across the paired-pulse protocols (Figure 3a). Post-hoc analyses within excitability protocols confirmed similar patterns for both muscles, with significant differences between ICF, SP, and SICI (ICF > SP, *p* < 0.001; SP > SICI, *p* < 0.001; ICF > SICI, *p* < 0.001) (Figure 3b). Moreover, ADM responses were overall greater than FDI (*p* = 0.001) (Figure 3c).

### Action observation

A three-way repeated measures ANOVA was performed on normalized motor evoked potentials (MEPs), with factors Movement (Index finger, Little finger, Neutral), Muscle (FDI, ADM), and Time (0, 320, 640 ms).

The analysis revealed a significant Movement × Muscle interaction [*F*(1.163, 30.238) = 5.55, MSE = 19,289.46, *p* = 0.021, η^2^ = 0.18], as well as a main effect of Muscle [*F*(1, 26) = 6.61, MSE = 41,606.12, *p* = 0.016, η^2^ = 0.20].

**Figure 4:**
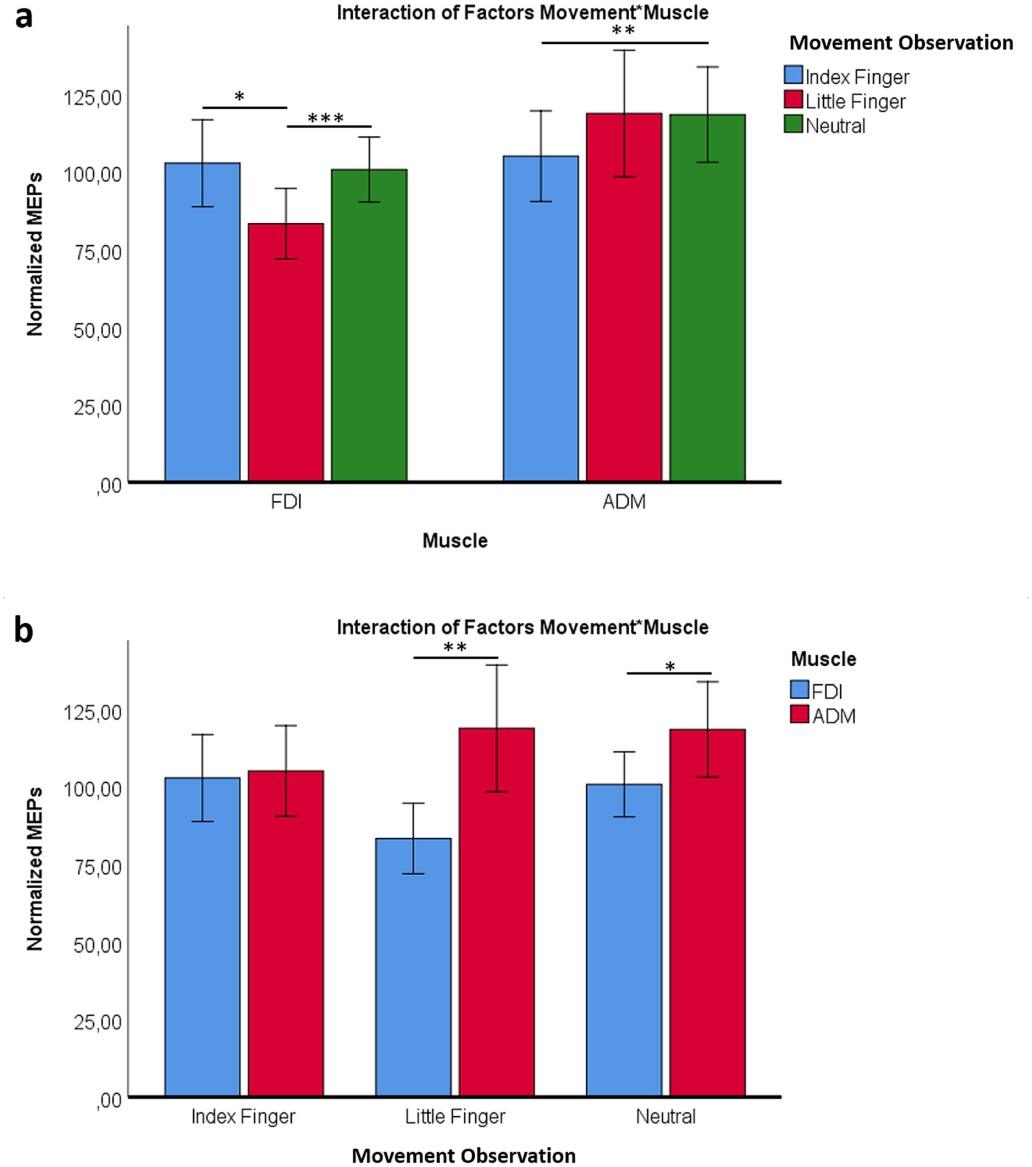
**(a)** Differences in MEP amplitudes between ADM and FDI as a function of movement type (index finger, little finger, neutral). **(b)** Differences in MEP amplitudes between movement conditions (index finger, little finger, neutral) shown separately for ADM and FDI. *p < 0.05; **p < 0.01; ***p < 0.001.

Post-hoc comparisons clarified these effects. The FDI muscle showed significant inhibition during observation of little finger movements compared to both index finger (*p* = 0.021) and neutral (*p* < 0.001) conditions. In contrast, the ADM muscle displayed higher excitability during neutral movements compared to the index finger (*p* = 0.005). Moreover, during little finger observation, ADM excitability was greater than FDI (*p* = 0.006) (Figure 4a).

Further inspection indicated that differences in the neutral condition were primarily driven by increased ADM excitability (*p* = 0.019). For the little finger condition, the significant contrast between ADM and FDI (*p* = 0.006) reflected both an excitatory increase in ADM and inhibition in the non-related FDI muscle (Figure 4b).

**Figure 5:**
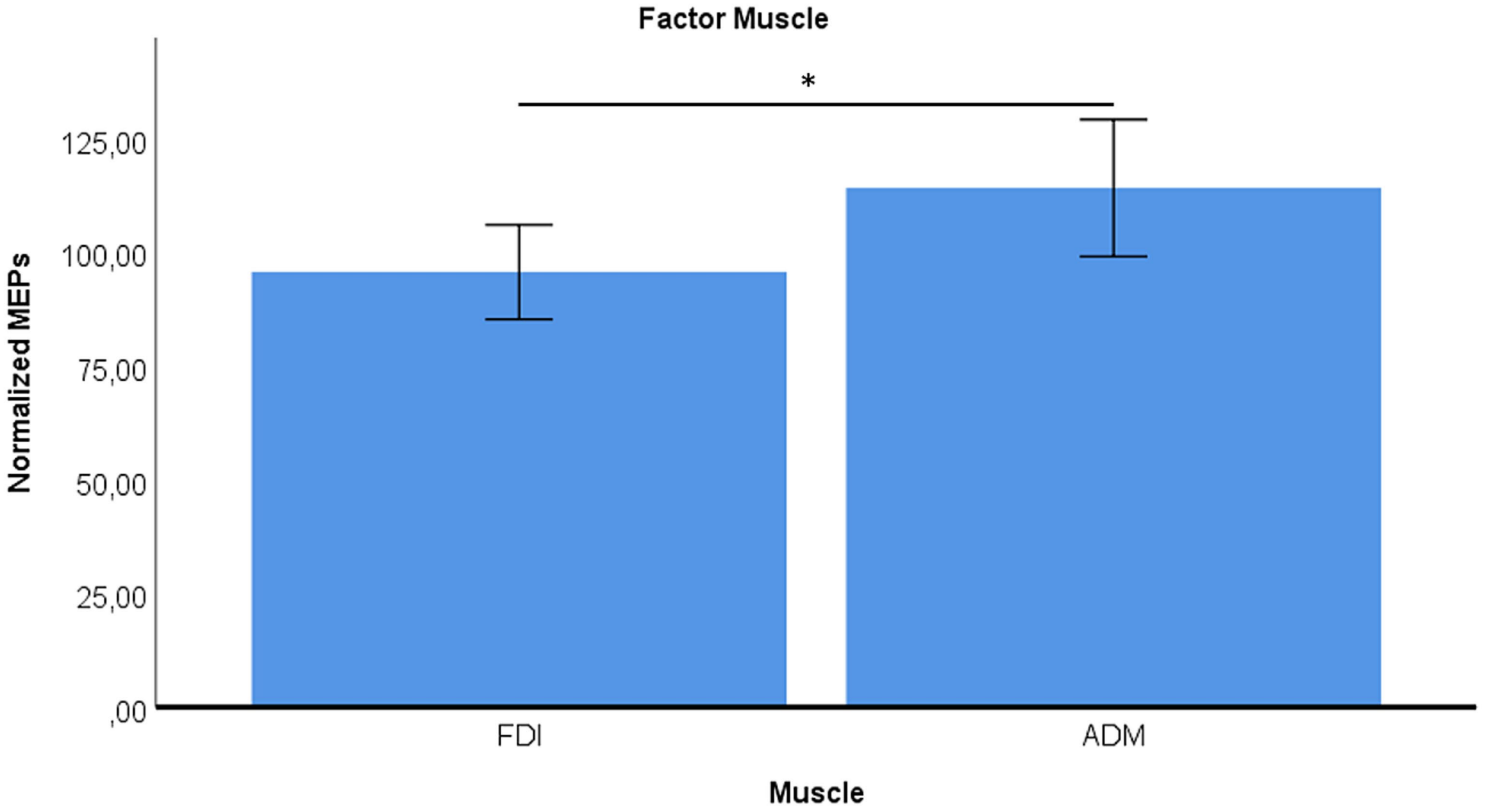
Overall difference in normalized MEP amplitudes between ADM and FDI across movement conditions. *p < 0.05.

Finally, across all conditions, ADM exhibited generally higher excitability than FDI (main effect of Muscle, *p* = 0.016) (Figure 5).

### Correlation between excitability protocols and action observation

We performed Pearson correlation analyses to examine the relationships between MEPs recorded from the ADM and FDI muscles during the observation of little finger and Index finger movements. These correlations were tested against excitability measures obtained from SICI and ICF protocols, both before and after the AO task. Since no significant effect of factor Time (time of stimulation) was found during the AO task (*p* = 0.913), MEPs from ADM and FDI were merged across time points for analysis.

**Figure 6:**
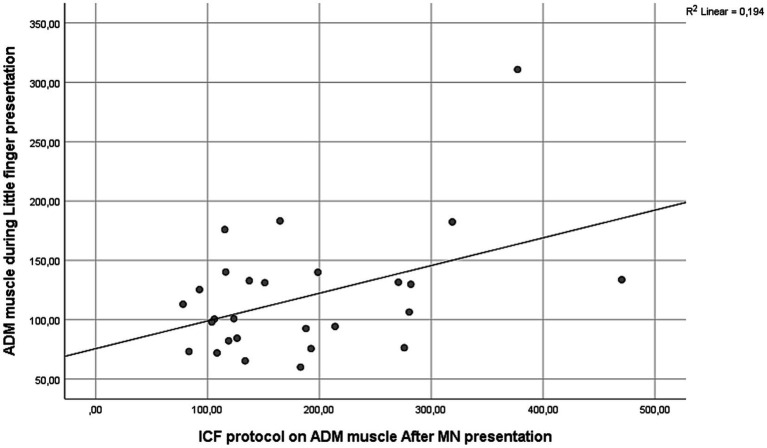
Moderate positive correlation between ADM MEPs during little finger movement observation and ICF measures of ADM obtained post-AO [*r*(25) = 0.441, *p* = 0.021].

**Figure 7:**
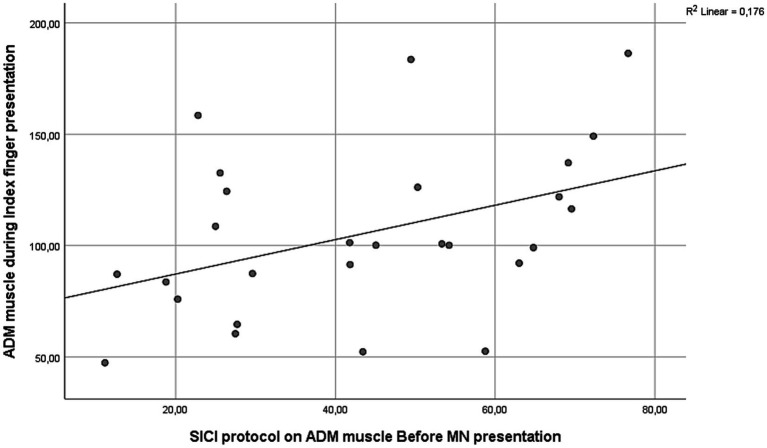
Moderate positive correlation between ADM MEPs during index finger movement observation and SICI measures of ADM obtained pre-AO [*r*(25) = 0.419, *p* = 0.029].

For the ADM muscle, a moderate positive correlation was found between excitability during little finger movement observation and ICF measured post-AO [*r*(25) = 0.441, *p* = 0.021], indicating that stronger facilitation after the AO task was associated with greater ADM activation during little finger movement observation (Figure 6). Additionally, ADM responses during index finger movement observation correlated positively with SICI measured pre-AO [*r*(25) = 0.419, *p* = 0.029], suggesting that higher inhibitory tone correlated with stronger inhibitory responses in ADM during index finger movement observation (Figure 7).

**Figure 8:**
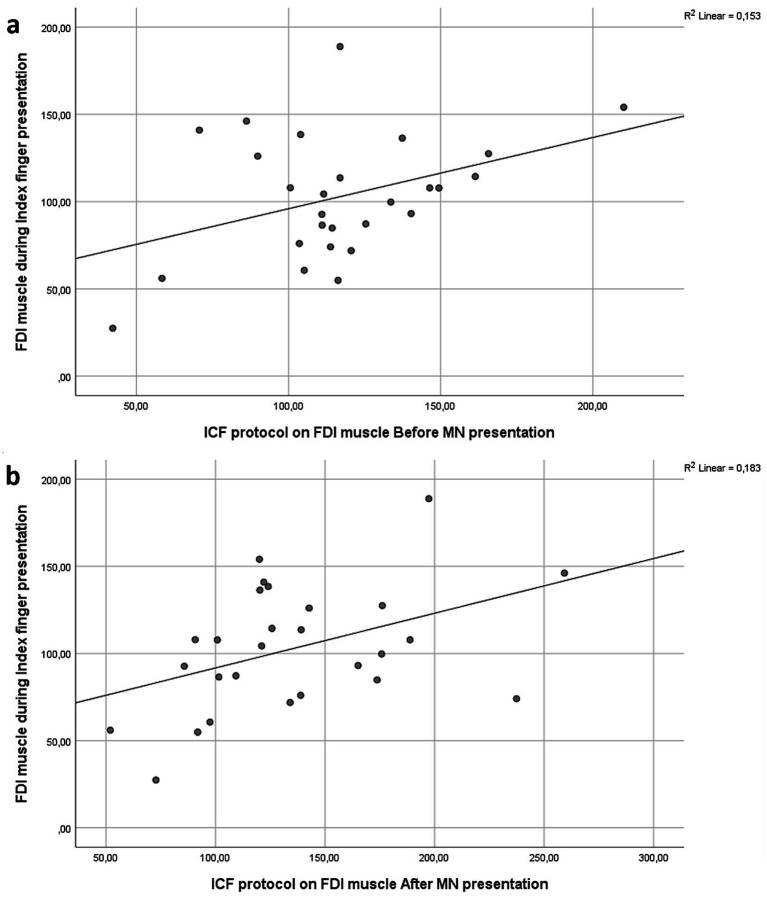
**(a)** Moderate positive correlation between FDI MEPs during index finger movement observation and ICF measures of FDI obtained pre-AO [*r*(25) = 0.428, *p* = 0.026]. **(b)** Moderate positive correlation between FDI MEPs during IF movement observation and ICF measures of FDI obtained post-AO [*r*(25) = 0.391, *p* = 0.044].

For the FDI muscle, significant correlations emerged with ICF measures. Specifically, pre-AO ICF correlated positively with FDI excitability during index finger movement observation [*r*(25) = 0.428, *p* = 0.026], and post-AO ICF also showed a moderate positive correlation with FDI responses during index finger movement observation [*r*(25) = 0.391, *p* = 0.044], reflecting a consistent facilitation effect across time points (Figures 8a,b).

## Discussion

The present study shows muscle-specific modulation of corticospinal excitability during action observation, together with systematic effects of paired-pulse indices of cortical excitability. During the AO task, the FDI was inhibited when participants observed little finger movements, whereas ADM showed enhanced excitability, particularly during little finger and neutral conditions (Figure 4). Across excitability protocols, ICF produced the largest MEPs, SP was intermediate, and SICI produced the smallest responses, with ADM larger than FDI overall (Figures 2, 3). We also observed a session-wide increase in normalized MEPs after the AO block. Importantly, correlational analyses linked excitability indices to AO responses: higher ADM ICF after AO related to larger ADM MEPs during little finger observation; higher ADM SICI before AO related to stronger ADM inhibition during index finger observation; and FDI ICF, both before and after AO, related to FDI facilitation during index finger observation (Figures 6–8). Together, these findings indicate that motor resonance during AO is shaped by the balance of facilitation and inhibition indexed by ICF and SICI.

The AO effects observed here are consistent with current accounts of motor resonance as a selective engagement of task-relevant motor representations accompanied by parallel suppression of non-relevant effectors. Specifically, the inhibition of FDI during observation of little finger movements aligns with the notion of motor surround inhibition, a control mechanism that prevents spurious co-activation while a non-matching effector is represented (Sartori et al., 2013; Lepage et al., 2012; Vigneswaran et al., 2013; Concerto et al., 2015; Bell et al., 2018; Ragimova et al., 2024; Nieto-Doval et al., 2023).

Several non-exclusive mechanisms may account for this inhibitory pattern. One possibility is that it reflects competitive interactions between adjacent motor representations within primary motor cortex (M1), whereby facilitation of the representation corresponding to the observed effector is accompanied by concurrent suppression of incongruent representations. A closely related account is inhibitory sharpening, in which suppression of the non-relevant effector enhances the selectivity of motor resonance and reduces cross-activation of neighboring representations. Additionally, this inhibition may serve as an active control mechanism limiting covert imitation or nonspecific motor output when the observed movement is incongruent with the recorded muscle. This framework is consistent with our previous findings showing that action observation induces excitation in the matching muscle alongside inhibition in the non-matching muscle, with the clearest effector selectivity emerging when stimulation is delivered after movement completion (Nieto-Doval et al., 2025). Together, these interpretations support the view that FDI inhibition during incongruent observation reflects a structured inhibitory component of motor resonance rather than a global suppression of corticospinal excitability.

The larger ADM responses for little finger and neutral conditions may reflect attentional set and a readiness state within the corresponding representation, in line with prior work on AO timing and presentation format, including our own recent findings that post-movement sampling yields the strongest modulation (Ragimova et al., 2024; Nieto-Doval et al., 2023).

An asymmetry was observed between FDI and ADM responses, with more robust and temporally consistent modulation in FDI despite identical stimulation parameters. This pattern likely reflects intrinsic differences in motor system organization rather than unequal stimulation efficacy. The index finger has a larger and more finely differentiated cortical representation and plays a central role in individuated, goal-directed actions, which may render FDI more sensitive to both facilitatory motor resonance and inhibitory sharpening during action observation (Takahashi et al., 2005; Schabrun and Ridding, 2007; Hehl et al., 2022). Consistent with this interpretation, our previous work demonstrated that inhibition of non-matching muscles during action observation is more reliably expressed in FDI, particularly when stimulation is delivered after movement completion (Nieto-Doval et al., 2025). Together, these findings suggest that the observed FDI–ADM asymmetry reflects functional specialization and differential inhibitory control across effectors, rather than a methodological artifact.

Previous work has demonstrated that corticospinal modulation during action observation is not merely epiphenomenal, but functionally relevant for motor learning and performance. In particular, facilitation of corticospinal excitability during action observation has been linked to improved imitation accuracy, enhanced motor learning, and greater training-induced plasticity (Buccino et al., 2001; Stefan et al., 2005; Celnik et al., 2006). From this perspective, motor resonance reflects a mechanism by which observed actions are mapped onto the observer’s motor system in a way that supports subsequent motor performance (Fadiga et al., 1995; Aglioti et al., 2008).

The present findings extend this literature by emphasizing that motor resonance is shaped not only by facilitation of task-relevant representations, but also by structured inhibition of non-relevant effectors. Such inhibitory shaping may be critical for refining observed action representations and preventing non-specific motor activation, thereby enhancing the fidelity of motor learning. Although no behavioral measures were collected here, the observed relationship between intracortical inhibitory tone and effector-specific suppression during action observation suggests a plausible pathway by which individual differences in excitation–inhibition balance could influence learning efficiency or performance outcomes. This interpretation aligns with prior work linking corticospinal modulation during action observation to functional motor gains, and highlights inhibitory mechanisms as a potentially underappreciated contributor to the behavioral relevance of motor resonance (Sohn and Hallett, 2004; Kassavetis et al., 2012; Aoyama et al., 2019).

The excitability-protocol results reproduced the expected physiological changes. SICI, a GABA-A-mediated measure, reduced MEPs; ICF, which depends on glutamatergic NMDA mechanisms, increased MEPs; and SP provided an internal reference within the same randomized blocks (Kujirai et al., 1993; Chen et al., 2018; Rothwell et al., 2009; Cash et al., 2017; Van den Bos et al., 2018; Leodori et al., 2019; Ibáñez et al., 2020). The correlations bridge these physiological indices with AO responses at the muscle level. ADM ICF after AO tracked ADM facilitation during little finger observation, suggesting that a more excitable glutamatergic state was expressed in the effector relevant to the viewed movement. ADM SICI measured before AO tracked ADM inhibition during index finger observation, consistent with a stronger inhibitory set being expressed when the observed action did not match the ADM representation. Because index finger movements are incongruent with the ADM effector, suppression of ADM activity during their observation can be interpreted as an inhibitory gating mechanism that prevents spurious activation of non-matching motor representations (Sohn and Hallett, 2004). The fact that the magnitude of this suppression scaled with resting SICI suggests that individual differences in baseline GABAergic inhibitory tone constrain how strongly non-relevant effectors are downregulated during action observation. In this view, SICI does not merely index generic inhibition at rest, but reflects a functional inhibitory set that sharpens motor resonance and limits covert imitation or overflow activation during observation of incongruent actions. FDI ICF, both before and after AO, tracked FDI facilitation during index finger observation, indicating that excitatory drive in the corresponding representation promotes the amplitude of motor resonance. These converging associations support the view that the magnitude and sign of motor resonance depend on the local balance between glutamatergic facilitation and GABAergic inhibition in M1 and connected premotor circuits. Beyond describing muscle-specific associations, these correlations provide a mechanistic link between static measures of intracortical excitability and dynamic corticospinal responses during AO. SICI and ICF are typically assessed at rest and index relatively stable inhibitory and facilitatory properties of M1 (Stagg et al., 2011). The present findings indicate that interindividual differences in these baseline excitation–inhibition profiles systematically constrain how strongly motor representations are facilitated or suppressed during AO, consistent with recent evidence distinguishing trait-like from state-dependent inhibitory control (Nuara et al., 2023). In this sense, paired-pulse excitability measures bridge canonical physiological indices with the expression of motor resonance, helping to explain variability across muscles and individuals (Enticott et al., 2012; Naish et al., 2014).

We also noted an overall increase in normalized MEPs across the session. The intensity used for test pulses, 120% rMT, likely recruited both direct and indirect corticospinal volleys, and the accumulated stimulation may have induced LTP-like processes, which can elevate MEPs later in the session (Suppa et al., 2022). At the same time, the AO effects were movement- and muscle-specific and all analyses were normalized to the single Baseline recorded before any AO, arguing against a purely nonspecific arousal account. Intensity-dependent map scaling (the phenomenon whereby increasing TMS intensity expands the effective cortical area activated by stimulation, resulting in broader recruitment of corticospinal neurons and, consequently, larger and less focal MEP responses) may additionally contribute to greater ADM percentages at 120% rMT, given known changes in cortical map area and recruitment with higher intensities (van de Ruit and Grey, 2016; Malcolm et al., 2006; Nazarova et al., 2021).

Limitations and methodological considerations merit mention. First, intensity choice matters for AO studies. Lower test intensities around 100–110 percent rMT can be more sensitive to motor resonance while higher intensities increase direct corticospinal recruitment that may mask subtle AO effects (Loporto et al., 2013; Cracco et al., 2016; Burgess et al., 2013). Second, because the TMS hot-spot favored a larger FDI at Baseline, percent normalization can inflate relative changes in ADM when absolute Baseline amplitudes differ between muscles. To address this concern, absolute MEP amplitudes are provided in Supplementary material. Third, several methodological considerations should be noted. SICI and ICF were assessed using a single interstimulus interval and a single conditioning intensity. While this approach is sufficient to capture relative inhibitory and facilitatory modulation, sampling multiple ISIs and conditioning intensities would allow future studies to more fully characterize the inhibitory and facilitatory profiles of intracortical circuits. In addition, we did not acquire input–output (I–O) functions (recruitment curves), which describe how MEP amplitude scales across stimulation intensities and can help distinguish changes in recruitment dynamics from changes in overall corticospinal gain. These aspects are.

therefore, framed as recommendations for future work aimed at refining mechanistic interpretation, rather than limitations that compromise the primary conclusions of the present study. Fourth, attention was not explicitly manipulated, despite known effects on MEPs during AO (Bell et al., 2018). Fifth, although we observed session-wide changes in excitability indices, the present design did not include a post-task single-pulse baseline. Therefore, fatigue-related effects or cumulative stimulation effects were not formally tested analytically and cannot be dissociated from more general session-dependent changes in corticospinal excitability. Post-task measurements were limited to paired-pulse indices (SP, SICI, ICF), which were used to characterize overall shifts in excitatory–inhibitory balance rather than to rule out nonspecific factors. Finally, we did not include spinal or peripheral measures, so all inferences are cortical or cortico-cortical.

In summary, AO produced effector-specific facilitation and surround inhibition, and these responses were systematically related to paired-pulse indices of cortical inhibition and facilitation. Measuring SICI and ICF at rest and again after the task allowed us to separate trait-like from state-dependent influences, indicating that the local excitation-inhibition balance in M1 constrains both the sign and magnitude of motor resonance. The integrated design, combining within-subject paired-pulse profiling with an optimized post-movement sampling window, links neurotransmitter-specific excitability to corticospinal modulation during observation. This framework advances the field from descriptive effects toward mechanism and suggests SICI and ICF as candidate biomarkers for conditions with altered excitation-inhibition balance and atypical motor resonance. Clinically, coupling SICI and ICF with AO offers a mechanistic biomarker framework. In disorders with altered excitation-inhibition balance, such as focal dystonia (Porcacchia et al., 2019) and selected neuropsychiatric conditions, these measures could support phenotyping, predict responsiveness to AO or imitation-based therapies, and track target engagement for interventions that modulate GABAergic or glutamatergic transmission pharmacologically or via noninvasive brain stimulation. Furthermore, dysfunction in motor resonance mechanisms has been reported in several neuropsychiatric and motor disorders, such as autism spectrum disorders (Masuda et al., 2019; Chan and Han, 2020), schizophrenia (Mehta et al., 2014) and Parkinson’s disease (Farina et al., 2020), underscoring the clinical relevance of understanding the neural and neurochemical substrates of this phenomenon. Because the indices are obtainable within a single session, they are suited to longitudinal monitoring and dose optimization. Although the present data are from healthy adults, the protocol is readily transferable to patient cohorts and may help personalize stimulation parameters and rehabilitation timing.

## Data Availability

The original contributions presented in the study are included in the article/Supplementary material, further inquiries can be directed to the corresponding author.

## Glossary

ADM: abductor digiti minimi
AO: action observation
ANOVA: analysis of variance
EMG: electromyography
FDI: first dorsal interosseous
GABAA: gamma-aminobutyric acid type A
ICF: intracortical facilitation
IF: index finger
ISI: interstimulus interval
ITI: inter-trial interval
LF: little finger
MEP: motor evoked potential
M1: primary motor cortex
MRI: magnetic resonance imaging
MNS: mirror neuron system
MSE: mean squared error
r: Pearson correlation coefficient
rMT: resting motor threshold
SICI: short-interval intracortical inhibition
SP: single pulse
TMS: transcranial magnetic stimulation
