# Supplementary material for: GABAergic and glutamatergic mechanisms of cortical excitability shape motor resonance during action observation as measured by transcranial magnetic stimulation
Source: Front Hum Neurosci. 2026 Mar 23;20:1746409. doi: 10.3389/fnhum.2026.1746409 (PMC13050837; doi:10.3389/fnhum.2026.1746409)
Supplement: Supplementary file 1 [file Data_sheet_1.docx]

Files for **ICF, SICI, SP, and Action observation**:

Tab “Subjects Raw Data”: For each subject - Raw MEPs, Average and calculations for cleaning with 2SD, New average after cleaning

Tab “Before Exclusion”: For each subject – Average values before 2SD cleaning

Tab “After Exclusion”: For each subject – Average values after 2SD cleaning

Tab “Normalization”: Values normalized before and after 2SD cleaning

File **Correlations**:

For each subject, average normalized values: for each muscle on each excitability protocol before and after AO, and merged values (the three times of stimulation) for each muscle during each movement (index and little finger)

Table 1 – Action Observation

Table 2 – Correlation

Table 3 – ICF before and after AO

Table 4 - SICI before and after AO

Table 5 - SP before and after AO
